# *Mycobacterium tuberculosis* ESX-1-secreted substrate protein EspC promotes mycobacterial survival through endoplasmic reticulum stress-mediated apoptosis

**DOI:** 10.1080/22221751.2020.1861913

**Published:** 2021-01-17

**Authors:** Qinglong Guo, Jing Bi, Honghai Wang, Xuelian Zhang

**Affiliations:** a State Key Laboratory of Genetic Engineering, School of Life Science, Fudan University, Shanghai, People’s Republic of China; b National Clinical Research Center for Infectious Disease (Tuberculosis), Shenzhen Third People’s Hospital, South University of Science and Technology of China, Shenzhen, People’s Republic of China; c Key Laboratory of Medical Molecular Virology, Ministry of Education and Health, School of Basic Medical Sciences, Fudan University, Shanghai, People’s Republic of China; d Shanghai Engineering Research Center of Industrial Microorganisms, Fudan University, Shanghai, People’s Republic of China

**Keywords:** *M. tuberculosis*, ESX secretion-associated protein C, endoplasmic reticulum stress, caspase activation, mitochondria damage, macrophage apoptosis

## Abstract

EsxA, secreted by the ESAT-6 secretion system 1 (ESX-1) secretion system, is considered the major *Mycobacterium tuberculosis* (*Mtb*) virulence determinant. However, the roles of the individual ESX-1 substrates, such as EspC, remain unclear due to their interdependency for secretion with EsxA. Here, we validated that EspC triggered ER stress-mediated apoptosis in macrophages. The EspC-mediated ER stress was involved in pro-inflammatory cytokines generation, intracellular Ca_2+_ release, and reactive oxygen species accumulation. Mitochondrial transmembrane potential dissipation and mitochondrial outer membrane permeabilization occurred in EspC-treated macrophages, causing apoptosis. Furthermore, ER stress-mediated apoptosis was effectively induced in EspC-overexpressing Mycobacterium smegmatis-infected macrophages and mice. EspC overexpression caused a significant increase in bacterial survival in the macrophages, spleens, and lungs, and accelerated mouse death was observed. Moreover, the increased viability of bacteria in the macrophages was significantly reduced by pretreatment with the apoptosis inhibitor. Overall, our results revealed that EspC is an essential ESX-1 protein for *Mtb*–host interactions and EspC-induced ER stress-mediated apoptosis may be employed by Mtb to establish and spread infection. Given the critical roles of the ESX systems in *Mtb* pathogenesis and immunity, our findings offer new perspectives on the complex host-pathogen interactions and mechanisms underlying ESX-1-mediated pathogenesis.

## Introduction

*Mycobacterium tuberculosis* (*Mtb*), the primary causal agent of tuberculosis (TB), was responsible for 1.40 million deaths globally in 2019 [1]. On the one hand, as major effector cells, host macrophages can phagocytose *Mtb* and restrain its survival [2,3]. On the other hand, *Mtb*, one of the most successful human pathogens, can escape these host defense mechanisms by blocking phagosome maturation, mediating inflammatory responses and manipulating host cell death programs [4,5]. Successful infection requires the secretion of virulence factors, and the proteins secreted by the ESX-1 secretion system are vital for the virulence and pathogenicity of mycobacteria [6,7]. Particularly, EsxA, the best-known virulence protein from the ESX-1 secretion system of *Mtb*, has been extensively studied in host–pathogen interactions. EsxA leads to membrane disruption [8,9], autophagy inhibition [10], apoptosis induction [11], and the weakening of the host’s innate immune response by inhibiting toll-like receptor 2-nuclear factor-κB (TLR2-NF-κB) cascades and downmodulating the host’s adaptive immunity [12,13]. Thus, the experimental evidence ascribes a critical role for ESX-1-mediated *Mtb* pathogenesis through EsxA cytolytic activity and its downstream effects on the host. However, a recent study has suggested that ESX-1-mediated cell lysis occurs through a contact-dependent gross membrane disruption mechanism, thereby implying that there may exist other mechanisms for EsxA or other ESX-1-secreted effectors in host–pathogen interactions [14].

However, studying the exact role of each ESX-1 substrate is complicated as EsxA/EsxB secretion depends on the presence of several Esp proteins, and the deletion of *esxA/esxB* abolishes the secretion of different Esp proteins [15–18]. Particularly, EspC, encoded by an *espACD* cluster located more than 260 kb upstream of the *esx-1* locus, is an indispensable protein for the secretion of EsxA. In addition, *esxA* deletion mutants are unable to secrete EspC, which explains why EspC is not secreted in Bacillus Calmette–Guérin (BCG) despite the presence of *espC* in the BCG genome [15,17,18]. The *espACD* locus is highly conserved and restricted to pathogenic mycobacteria, including *Mycobacterium leprae*, which has a downsized genome [19]. Despite being a critical protein associated with EsxA secretion from the ESX-1 system and forming a filamentous structure in the cell envelope of *Mtb* [20], the interactions of EspC with host macrophages are not completely understood.

We have previously found that EspC activated macrophages and induced the secretion of pro-inflammatory cytokines through the TLR4-dependent mitogen-activated protein kinase (MAPK) signaling pathway [21]. In the present study, we demonstrate that EspC is another critical virulent factor from the *Mtb* ESX-1 system that mediates *Mtb*–macrophage interactions by triggering endoplasmic reticulum (ER) stress-mediated apoptosis and promoting mycobacterial infection.

## Materials and methods

### Mice and cell lines

C57BL/6 mice were obtained from the Animal Center of Slaccas (Shanghai, China). All mice were maintained under specific pathogen-free conditions in the Animal Center of the School of Life Science of Fudan University. All animal procedures were conducted in accordance with the Guide for the Care and Use of Laboratory Animals of the National Institutes of Health, and the study protocol was approved by the Animal Care and Use Committee of Fudan University. The RAW264.7 cell line was purchased from the American Type Culture Collection (Manassas, VA, USA). RAW264.7 cells were cultured in Dulbecco’s modified Eagle’s medium (DMEM; Gibco, Grand Island, NY, USA) supplemented with 10% fetal bovine serum (FBS), penicillin (100 U/mL), and streptomycin (100 mg/mL) and maintained at 37 °C in a humidified incubator (5% CO_2_).

### Cloning, expression, and purification of recombinant EspC and construction of Mycobacterium smegmatis::espc (Ms::espc)

The *espC* gene was cloned, expressed, and purified, and *Ms::espC* was constructed as described in our previous study [21]. Briefly, EspC expression was induced with isopropyl-β-D-1-thiogalactopyranoside (IPTG) for 12 h at 37 °C after the bacteria were grown to OD_600_ = 0.6–0.8. Then, the cells were harvested and ultrasonicated in PBS. After centrifugation, the precipitation was dissolved in the buffer containing 20 mM Tris (pH 8.0), 500 mM NaCl, 8 M urea, 5% glycerol, 10 mM imidazole, and 2 mM β-mercaptoethanol with a protease inhibitor cocktail and DNase I [23]. The N-terminal His-tagged recombinant EspC was purified using HIS-Select Nickel Affinity Gel (Sigma-Aldrich, St. Louis, MO, USA). The purified and denatured EspC proteins were dialyzed to remove urea for renaturation and filtered using a Sephadex G-75 chromatography column (GE Healthcare, Uppsala, Sweden) to remove other non-specific proteins. The dialyzed recombinant EspC was incubated with polymyxin B-agarose (Sigma) overnight at 4 °C to remove the endotoxins. Endotoxin content was determined to be extremely low (<0.05 EU/mg) as detected using an E-TOXATE kit (Limulus amebocyte lysate; Sigma-Aldrich). Here, Ag85A was chosen as the negative control[22], which was cloned, expressed, and purified, and the endotoxins were removed under the same conditions as those of EspC. Thapsigargin (TG), an inhibitor of the microsomal Ca^2+^-ATPase and a well-characterized ER stress-inducing agent [23], was used as the positive control.

To investigate whether the Ms::*espC* strain secretes EspC, the indicated strains were cultured in Sauton’s medium containing 30 µg/mL kanamycin for 12 h. The bacteria and the cell-culture supernatant were then harvested for EspC protein secretion analysis [24,25]. Ms::PSQ was the empty vector control strain. Ms::*esat-6* expressing and secreting ESAT-6 was used as the positive control. Equal amounts of the recombinant Ms::*espC* were incubated with proteinase K (100 µg/ml) at 37 °C at the indicated times. The activity of the proteinase K was terminated by the addition of 1 X complete EDTA-free protease inhibitor cocktail (Roche, Basel, Switzerland). Then, the cells were subjected to western blot using antibodies against His-tag and catalase-peroxidase gene (KatG) to analyze the expression of each protein. To determine the subcellular location of the EspC protein in *M. smegmatis* (*Msm*), the recombinant Ms::*espC* was cultured in Sauton’s medium and grown at the log-phase, and the bacteria were collected and sonicated. The lysates were centrifuged at 3 000 × g at 4 °C to precipitate the cellular debris and unlysed cells, whereas the supernatant was sedimented at 27 000 × g for 30 min to precipitate the cell-wall fraction and centrifuged again at 100 000 × g for 4 h to isolate the cytoplasmic membrane from the cytosolic fraction. Each fraction was subjected to western blotting using anti-His, anti-Ag85, and anti-GroEL1antibodies.

### Reagents

z-VAD-fmk was purchased from Biovision (Milpitas, CA, USA). 4-PBA, NAC, and BAPTA-AM were from Sigma Aldrich (St. Louis, MO, USA). IKK-2 IV was obtained from Santa Cruz Biotechnology (Santa Cruz, CA, USA). SP600125, U0126, and SB203580 were acquired from Cell Signaling Technology (Beverly, MA, USA). TG was purchased from Abcam (Cambridge, UK). RAW264.7 cells were pretreated with the indicated concentrations of the inhibitor for 1 h before EspC stimulation.

### Isobaric tags for relative and absolute quantitation (iTRAQ)-based quantitative proteomics analysis

RAW264.7 cells were seeded in 6-cm tissue culture plates and stimulated with or without 5 μg/mL EspC for 24 h at 37 °C and 5% CO_2_. The cells were harvested and re-suspended in 400 μL lysis buffer (7 M urea, 2 M thiourea, 2% CHAPS, proteasome inhibitor) and then ultrasonically crushed to extract the total proteins. Protein peptides (100 μg) from each group were labeled using the 8 plex iTRAQ reagents multiplex kit (ABI, Foster City, CA, USA). The reconstituted peptides were analyzed using a Q-Exactive mass spectrometer (Thermo Fisher Scientific, Waltham, MA, USA) coupled with a nano high-performance liquid chromatography system (UltiMate 3000 LC Dionex; Thermo Fisher Scientific). The peptide data were analyzed using the Proteome Discoverer 1.4 (v1.4.0.288; Thermo Fisher Scientific). Protein probabilities were estimated using the Protein Prophet algorithm, and proteins with at least two unique peptides were identified. The upregulated or downregulated proteins in both replicates with relative quantification *p*-values < 0.05 and 1.3-fold changes were determined as differentially expressed.

### Apoptosis analysis

Apoptotic cells were detected using an annexin V/propidium iodide (PI) staining kit according to the manufacturer’s instructions (BD Pharmingen Inc., San Diego, CA, USA). The cells were stained with FITC-conjugated annexin V and PI. The stained cells were analyzed using FACSCalibur (BD Biosciences, San Jose, CA, USA), and the data were processed using the Flow Jo 7.6.1 software.

### Cytokine measurement using ELISA

Pro-inflammatory cytokines (TNF-α, IL-6, and MCP-1) secreted by the macrophages in the culture supernatants were measured using sandwich enzyme-linked immunosorbent assay (ELISA) kits according to the manufacturer’s instructions (Biolegend, San Diego, CA, USA). Cytokine levels were determined based on the absorbance at 450 nm measured using a microplate reader.

### RNA extraction and real-time PCR

RAW264.7 cells were treated with 5 µg/ml EspC or Ag85A for 24 h or infected with Ms::PSQ or Ms::*espC* (MOI=10) for 24 h, following which the cells were collected and the total RNA was extracted using Trizol reagent (Invitrogen, Carlsbad, CA, USA) according to manufacturer’s instructions. The purity and the concentration of the RNA were determined using a spectrophotometer (Nanodrop 2000, Wilmington, DE, USA). Total RNA (1000 ng) was reverse transcribed using a PrimeScript® RT Reagent Kit with gDNA Eraser (Takara, Dalian, China). The cDNA was used for quantitative real-time PCR (qPCR) analysis on Applied Biosystems 7500 (Applied Biosystems, Foster City, CA, USA) with a SYBR® Green PCR Kit (Vazyme, Nanjing, China). All samples were analyzed in triplicate. The primers used in qPCR are shown in Table S4. The mRNA levels were normalized to those of GAPDH of the same cDNA sample. Relative quantification of gene expression was calculated using the 2^-ΔΔCt^ method.

### Western blot analysis

Anti-CHOP, anti-caspase-12, anti-caspase-9, anti-caspase-3, anti-caspase-3 cleaved, anti-Bip, anti-p-eIF2α, anti-p-JNK, anti-p-ERK, anti-p-p38, anti-IRE-1α, anti-Cyt C, and anti-COX IV antibodies were purchased from Cell Signaling Technology; anti-α-tubulin antibodies were acquired from Biyuntian (Nanjing, China); anti-Ag85 and anti-GroEL1 were obtained from Abcam (Cambridge, UK); and anti-BAX antibodies were from Santa Cruz Biotechnology. Goat anti-mouse-IgG-HRP (Proteintech, Wuhan, China) and goat anti-rabbit-IgG-HRP (Proteintech) were used as secondary antibodies. Western blots were detected using the Amersham^TM^ ECL^TM^ Prime western blotting detection reagent (GE Healthcare, Buckinghamshire, UK) and the ChemiScope 3400 mini imaging system (Clinx Science Instruments, Shanghai, China). The results shown are representative of three independent experiments.

### ROS measurement using flow cytometry

EspC-stimulated RAW264.7 cells were harvested and washed with PBS. To determine the intracellular hydrogen peroxide levels, cells were stained with DCFH-DA (10 µM; Sigma Aldrich), whereas for the intracellular superoxide levels, cells were stained with dihydroethidium (DHE; 20 µM; Sigma-Aldrich) both at 37 °C for 30 min in the dark. The cells were then washed with PBS and analyzed on FACSCalibur. Data were processed using FlowJo 7.6.1 software.

### Ca^2+^ measurements using FACS

Intracellular calcium concentrations were detected using a fluorescent calcium indicator, fluo-3/AM (Sigma Aldrich). RAW264.7 cells were washed twice with PBS and incubated with 5 µM fluo-3/AM for 30 min in Hank’s balanced salt solution (HBSS) containing 1 mM Ca^2+^, 1 mM Mg^2+^, and 1% FBS. After staining, the cells were washed twice with PBS, and the final pellets were resuspended with 1 mL HBSS containing 1 mM Ca^2+^, 1 mM Mg^2+^, and 1% FBS. Intracellular Ca^2+^ was measured using FACSCalibur, and data were processed using FlowJo 7.6.1 software.

### Assessment of mitochondrial membrane potential (ΔΨm)

ΔΨm was evaluated by measuring the retention of the lipophilic cationic dye DIOC_6_ (Sigma Aldrich) in the mitochondria. Cells were harvested and incubated in DIOC_6_ solution (40 nM in fresh medium) for 20 min at 37 °C in the dark. After incubation, the cells were washed once with PBS and resuspended in PBS. ΔΨm was assessed using a flow cytometer (Becton Dickinson), and the data were processed using FlowJo 7.6.1 software.

### Immunofluorescence microscopy

RAW264.7 cells were plated overnight on 20-mm glass-bottomed cell culture dishes. After treatment with EspC, the cells were incubated with the pre-warmed medium containing 250 nM of MitoSpy Red CMXRos (Biolegend) for 20 min. After the cells were washed with PBS, they were fixed and permeabilized in Immunol Staining Fix solution (Biyuntian) for 20 min and blocked with 3% bovine serum albumin (BSA) in PBS. The cells were washed with PBS and incubated with anti-cytochrome c and anti-Bax for 2 h at 37 °C in the Immunol staining primary antibody dilution buffer (Biyuntian). After the cells were washed with PBS, they were re-stained with the appropriate Alexa Fluor 488-conjugated secondary antibodies for 1 h at 37 °C and stained with 0.1 µg/mL DAPI for 10 min at 25 °C. The cells were analyzed using a Leica TCS SP8 laser scanning confocal microscope (Leica Microsystems, Wetzlar, Germany) (63X; NA, 1.4) oil immersion lens (HC PL APO CS2; zoom, 2.5X; speed, 400 Hz; line average and resolution, 4). Images were acquired and processed using the LAS AF Lite software.

### Mitochondrial and cytosolic fractionation

Mitochondrial and cytosolic proteins were isolated using the Mitochondria/Cytosol Fractionation kit (Beyotime, Nanjing, China) according to the manufacturer’s protocol. Cells (5 × 10^7^) were harvested, washed with ice-cold PBS, incubated with 1.0 mL cytosol extraction buffer mix provided in the kit for 15 min, and then homogenized using ice-cold Dounce tissue grinders. The homogenates were centrifuged at 600 × g for 10 min, and the supernatants were further centrifuged at 11 000 × g for 10 min at 4 °C. The cytosolic supernatants were decanted, and the pellets resuspended in 0.1 mL mitochondrial extraction buffer mix. Protein concentrations were determined using the BCA protein quantification assay with BSA as the protein standard.

### Intracellular infection assays

RAW264.7 cells were infected with Ms::*espC* and Ms::PSQ as previously described [21]. At the indicated times after infection, the cells were collected and subjected to flow cytometry to assess apoptosis and ΔΨm or western blotting with specific antibodies for each target protein.

### Mouse infection with Msm

*Msm* cultures grown to the mid-log phase were washed and resuspended in PBS. Mice were infected with *Msm* through the tail vein either at 1 × 10^8^ CFU/100 µL for mouse survival experiments or 1 × 10^7^ CFU/100 µL for tissue CFU detection, hematoxylin and eosin staining, and immunohistochemistry staining. The spleen and lung tissues from infected mice were homogenized in 1 mL PBS, diluted, plated on Middlebrook 7H10 agar supplemented with 10% OADC enrichment (both from Becton Dickinson), and incubated at 37 °C. Colonies were counted after 3 days. Some spleen samples were fixed in 4% paraformaldehyde, embedded in paraffin, sectioned, and stained with hematoxylin and eosin or subjected to immunohistochemistry staining of CHOP, Bip, and cleaved caspase-3 with the specific antibodies.

#### Statistical analysis

Data are expressed as the mean ± SEM (*n* = 3). Statistical significance was determined using unpaired *t*-test, one-way ANOVA followed by Tukey’s test, or two-way ANOVA followed by Bonferroni’s test, unless otherwise indicated. For the mouse survival study, Kaplan–Meier survival curves were generated and analyzed using a Gehan–Breslow–Wilcoxon test. Data analysis was performed in GraphPad Prism 5.0. *P* ≤ 0.05 was considered significant.

## Results

### Cellular proteome in EspC-stimulated macrophages

Recent studies have shown that purified EspC forms an SDS-resistant polymer that turned into monomers when treated with reducing agents [20] and that EspC is a thermally unstable protein with an α-helical structure [26]. Here, HIS-tagged EspC was overexpressed and purified in an *E. coli* expression system. The purified and homogenized proteins formed bands at approximately 10,000 MW and reacted with the anti-His antibody (Fig S1A–C).

Our previous studies have indicated that EspC interacts with TLR4 directly and initiates the production of pro-inflammatory cytokines by triggering the MAPK signaling pathway [21]. To further understand how EspC regulates the host immune response, RAW26.7 cells were incubated with EspC protein or solvent control for 24 h, and the proteins associated with EspC functions in macrophages were then investigated by iTRAQ. We found 98 differentially expressed proteins, 84 of which were upregulated and 14 were downregulated (Tables S1, S2). Many significantly differentially expressed proteins are involved in multiple processes, including response to stress, the MAPK/NF-κB signaling pathway, and apoptosis (Table S3). Further analysis revealed that many proteins involved in the ER stress response and oxidative stress were upregulated (Table S3). The ER has a vital role in folding secretory and cellular proteins during their transit, and cellular disturbances cause misfolded/unfolded proteins to accumulate in the ER [27], which is referred to as ER stress. Mycobacterial infection can cause apoptosis mediated by interactions between uncontrolled ER stress and ROS [27–29]. Accordingly, we hypothesize that EspC might triggers ER stress-mediated apoptosis.

### Espc stimulation induces ER stress-mediated macrophage apoptosis

**Figure 1.**
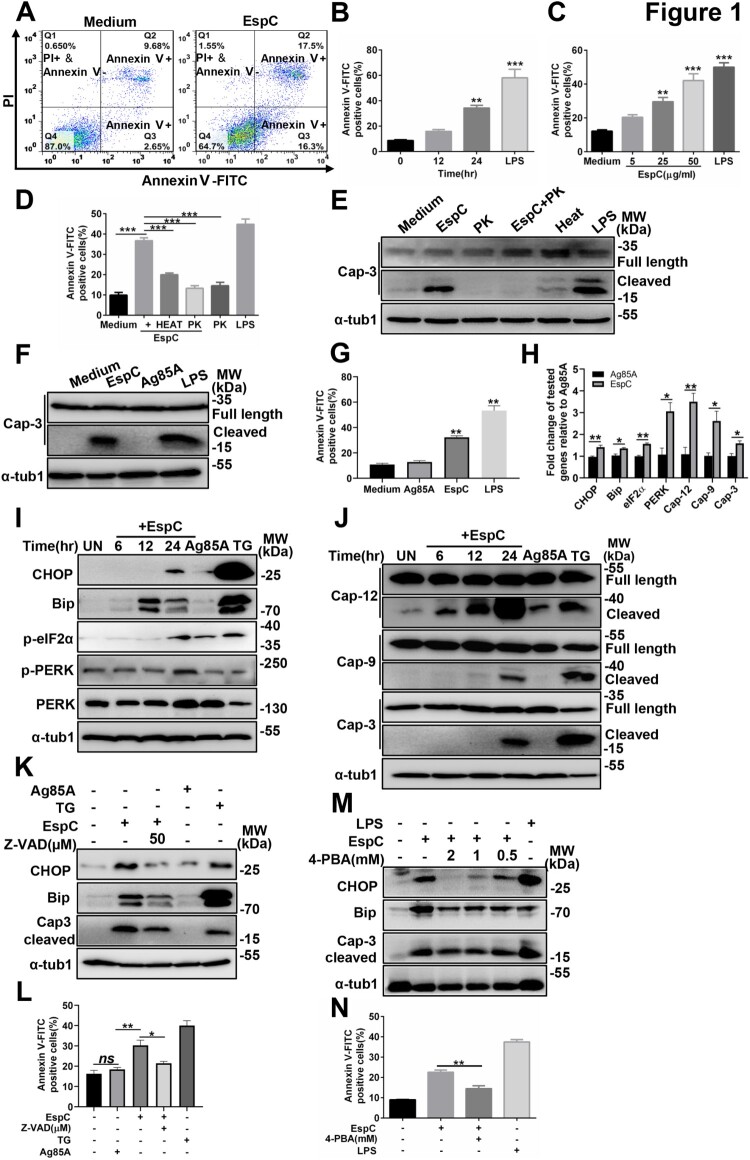
EspC stimulation induces ER stress-mediated apoptosis. **(A, B, C)** RAW264.7 cells were treated with 5 µg/mL EspC recombinant protein for various durations **(A, B)** or different concentrations of EspC for 24 h **(C)**. Next, the cells were collected and subjected to flow cytometry analysis. **(D, E)** RAW264.7 cells were incubated with 5 µg/mL EspC, proteinase K- or heat-treated EspC (5 µg/mL), proteinase K alone, and LPS for 30 h. Cells were collected, and apoptosis was analyzed using flow cytometry and western blot. (**F, G**) RAW264.7 cells were incubated with 5 µg/mL EspC or Ag85A for 30 h. Cells were collected, and apoptosis was analyzed using flow cytometry and western blot. **(H)** Real-Time PCR analysis of the indicated genes in EspC or Ag85A (5 µg/mL)-treated RAW264.7 cells. **(I)** Immunoblot analysis of CHOP, Bip, p-eIF2α, PERK, p-PERK, and α-tubulin (α-tub1) in the lysates of RAW264.7 cells stimulated with EspC or Ag85A (5 µg/mL) for 0–24 h in a time-dependent manner. **(J)** RAW264.7 cells were treated with EspC or Ag85A (5 µg/mL) for varying durations, and cell lysates were examined using western blot analysis with antibodies against caspase-12 (Cap-12), caspase-9 (Cap-9), caspase-3 (Cap-3), and α-tub1. **(K, L)** RAW264.7 cells were pretreated with Z-VAD-fmk for 1 h following EspC or Ag85A stimulation for 24 h. **(K)** Total cell lysates were analyzed using western blot analysis with antibodies against each target protein. **(L)** Cells were collected and subjected to flow cytometry for apoptosis analysis. **(M, N)** RAW264.7 cells were pre-incubated with 4-PBA for 1 h followed by EspC stimulation for 24 h. **(M)** Total cell lysates were analyzed using western blot analysis with antibodies against each target protein. **(N)** Cells were collected and subjected to flow cytometry for apoptosis analysis. Ag85A and TG were used as the unrelated negative and positive controls, respectively. LPS treatment was used as the positive control to induce ER stress. Data are shown as the mean ± SEM (*n* = 3); * *p* < 0.05, ** *p* < 0.01, *** *p* < 0.001.

To further characterize the modulatory function of EspC in macrophages and verify the above hypothesis, we first confirm whether EspC could induce macrophage apoptosis. The percentage of necrotic cells in EspC-treated macrophages was very low (1.55%), thereby excluding the possibility of EspC-induced necrosis (Figure 1A). In contrast, that of EspC-induced apoptotic cells was high (33.8%) (Figure 1A). EspC proteins induced cell apoptosis in a time- and dose-dependent manner (Figure 1B, C). Furthermore, heat and proteinase K treatments inhibited the apoptosis-inducing ability of EspC (Figure 1D, E), indicating that apoptosis did not result from the contamination of LPS in purified EspC. Recombinant Ag85A protein (negative control) did not induce apoptosis (Figure 1F, G).

In addition, we found that EspC upregulated the transcription of ER-stress markers, including CHOP, Bip, eIF2α, and protein kinase RNA- like ER kinase (PERK) (Figure 1H), the levels of CHOP and Bip proteins, and the phosphorylation of eIF2α and PERK in the macrophages compared with equal quality or molar of Ag85A treatment (Figure 1I, S2A). These results suggest that EspC could induce protein unfolding in the ER, thus resulting in ER stress.

ER stress mediates apoptosis in *Mtb* infection, and that is correlated with caspase activation [28,30]. Hence, we detected caspase-12, caspase-9, and caspase-3 activation in RAW264.7 cells after EspC treatment. The results showed that EspC treatment induced the gene expression and activation of caspases (Figure 1H, J, S2B) in comparison with Ag85A. Pretreatment with z-VAD-fmk, a pan-caspase inhibitor, reduced the level of EspC-induced CHOP and Bip (Figure 1K). The EspC-elicited activation of caspase-3 and cell apoptosis were decreased by z-VAD-fmk treatment (Figure 1K, L). Enhanced ER folding capacity and alleviated ER stress following 4-phenylbutyric acid (4-PBA) pretreatment could decrease the level of CHOP, Bip, and cleaved caspase-3 and lowered EspC-induced apoptosis, suggesting that EspC-mediated apoptosis was reduced by improving ER-folding capacity, thereby attenuating ER stress (Figure 1M, N). These results indicated that EspC-induced macrophage apoptosis is mediated by ER stress, which is closely related to caspase activation and the accumulation of unfolded or misfolded proteins in the ER.

### EspC-mediated ER stress is associated with the generation of pro-inflammatory cytokines

**Figure 2.**
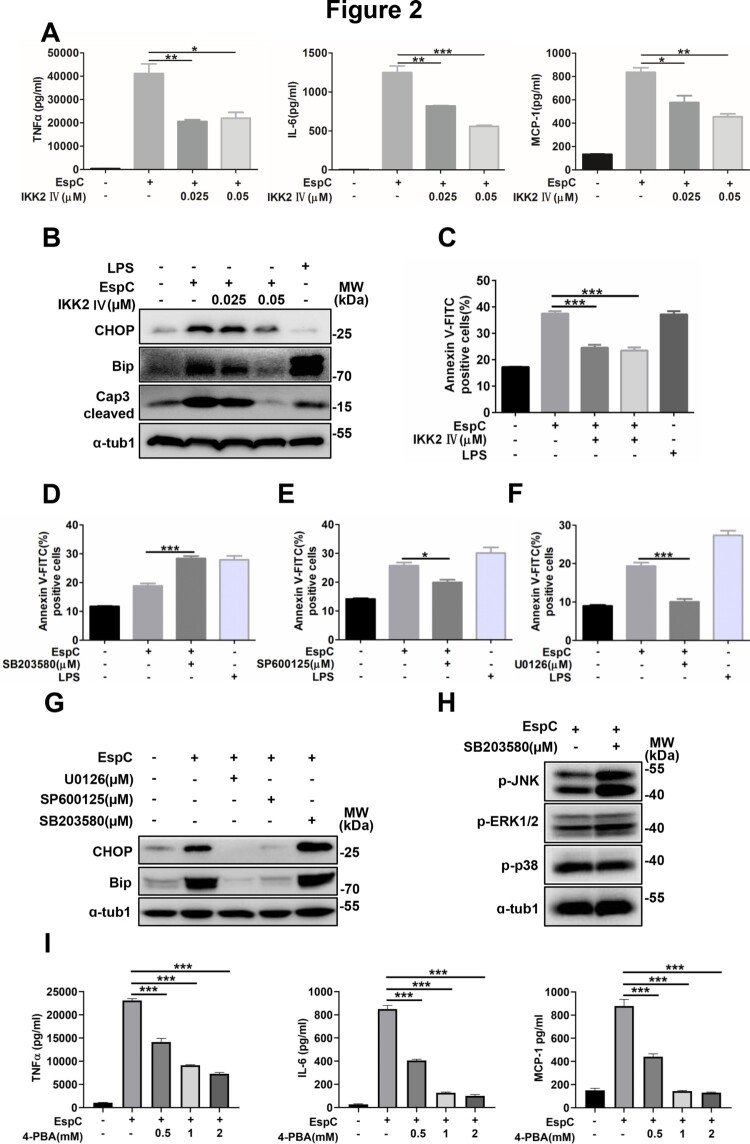
**EspC-mediated ER stress is associated with the generation of pro-inflammatory cytokines. (A–C)** RAW264.7 cells were pretreated with the IKK-2 inhibitor IKK-2 IV for 1 h before EspC stimulation. **(A)** The levels of pro-inflammatory cytokines (TNFα, IL-6, MCP-1) in the supernatant were measured using ELISA. **(B)** Cells were harvested, and total cell lysates were subjected to western blotting for each target protein. **(C)** The collected cells were subjected to flow cytometry to determine apoptosis after 30-h incubation with EspC (5 µg/mL). **(D–F)** RAW264.7 cells were pretreated with specific inhibitors—SB203580 **(D)**, SP600125 **(E)**, and U0126 **(F)**—for 1 h following EspC stimulation for 24 h, and then, the percentage of apoptotic cells was analyzed using flow cytometry. **(G)** Immunoblot analysis of CHOP, Bip, and α-tub1 in EspC-stimulated RAW264.7 cells pre-incubated with pharmacological inhibitors, p38 (SB203580; 10 µM), JNK (SP600125; 25 µM), and ERK (U0126; 10 µM), for 1 h. **(H)** MAPK pathway activation in EspC-treated RAW264.7 cells in the presence or absence of SB203580. **(I)** RAW264.7 cells were pretreated with 4-PBA for 1 h before EspC stimulation, and the levels of pro-inflammatory cytokines (TNFα, IL-6, MCP-1) in the supernatant were measured using ELISA. LPS treatment was used as the positive control to induce ER stress. Data are shown as the mean ± SEM (*n* = 3); * *p* < 0.05, ** *p* < 0.01, *** *p* < 0.001.

Our previous study have showed that EspC stimulation induced the production of pro-inflammatory cytokines, such as TNFα, IL-6, and MCP-1, via TLR4 signaling, all of which contain consensus-binding sites for NF-κB [21]. Here, we found that EspC-induced increases in TNFα, IL-6, and MCP-1 levels significantly decreased in RAW264.7 cells after pretreatment with an IκB kinase-2 (IKK-2) inhibitor in a dose-dependent manner (Figure 2A). Moreover, EspC-stimulated increases in CHOP and Bip levels were attenuated after pre-incubation with the IKK-2 inhibitor (Figure 2B), and caspase-3 activation and apoptosis caused by EspC were also reduced (Figure 2B, C).

EspC-induced increases in TNFα, IL-6, and MCP-1 levels were suppressed to varying degrees in cells preincubated with MAPK inhibitors, including SP600125 (c-Jun NH 2-terminal kinase (JNK) inhibitor), SB203580 (p38 inhibitor), and U0126 (Extracellular signal-regulated protein kinases (ERK) inhibitor) [21]. We further found that SB203580 treatment prominently increased EspC-induced apoptosis (Figure 2D). Conversely, JNK inactivation and the ERK inhibitor decreased EspC-induced apoptosis (Figure 2E, F). Additionally, the EspC-elicited production of CHOP and Bip was inhibited by SP600125 and U0126 (Figure 2G). However, pretreatment with SB203580 following EspC stimulation moderately increased CHOP and Bip expression compared with cells incubated with EspC alone (Figure 2G). SB203580 could also inhibit the activity of phosphatase and thus increase JNK phosphorylation and its activity (Figure 2H)[31], resulting in increased ER stress-mediated apoptosis. These data support that accelerated production pro-inflammatory cytokines via NF-κB and MAPK activation can lead to the accumulation of unfolded or misfolded proteins in the ER [44].

The disruption of ER homeostasis has also been reported to trigger the activation of NF-κB and MAPK, which further promotes the production of pro-inflammatory cytokines [32]. Here, we tested that whether EspC-induced ER stress has an effect on pro-inflammatory cytokine generation. We found that EspC-elicited generation of TNFα, IL-6, and MCP-1 was significantly decreased when the ER folding capacity improved following 4-PBA pretreatment (Figure 2I). Thus, we proposed that the EspC-induced generation of pro-inflammatory cytokines through TLR4-NF-κB/MAPK signaling initiates the accumulation of unfolded/misfolded proteins and ER stress. In turn, the EspC-induced ER stress further activated NF-κB and MAPK signaling and produced more pro-inflammatory cytokines.

### EspC-stimulated increase in intracellular Ca^2+^ levels is important in the ER stress response

**Figure 3.**
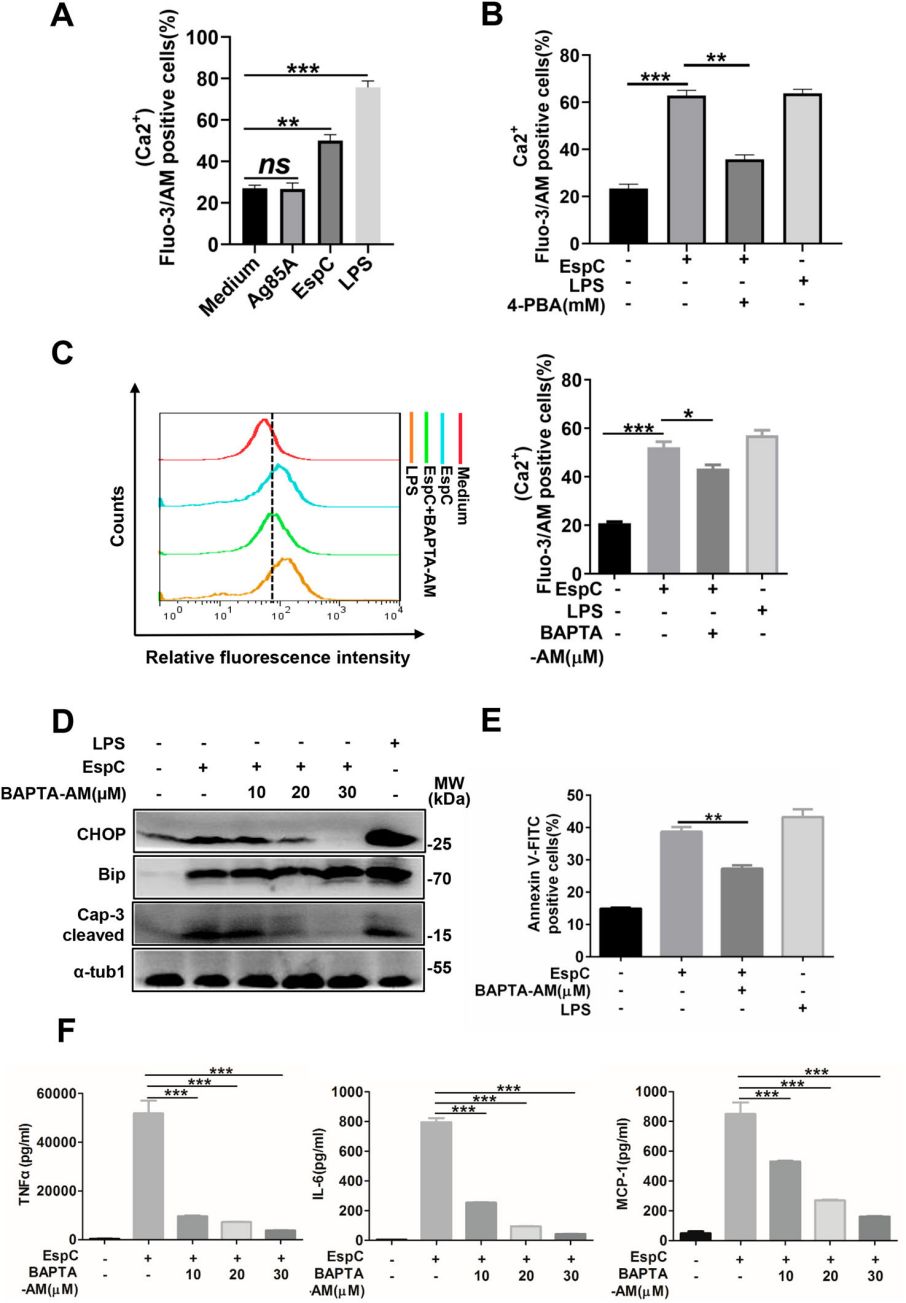
**EspC-stimulated increase in intracellular Ca^2+^ levels is important in the ER stress response. (A–C)** RAW264.7 cells were stimulated with EspC or Ag85A for 24 h **(A)**; or RAW264.7 cells were pretreated with or without 4-PBA or BAPTA-AM for 1 h, followed by EspC stimulation for 30 h **(B, C)**. The percentage of increase in intracellular Ca^2+^ levels was measured with fluo-3/AM using flow cytometry. **(D–F)** RAW264.7 cells were pretreated with BAPTA-AM for 1 h following incubation with EspC for 30 h. **(D)** Total cell lysates were examined using western blot analysis with specific antibodies against each target protein. **(E)** Cells were harvested for apoptosis analysis using flow cytometry. **(F)** The levels of pro-inflammatory cytokines (TNFα, IL-6, MCP-1) in the supernatant were detected using ELISA. LPS treatment was used as the positive control to induce ER stress. Data are shown as the mean ± SEM (*n* = 3); * *p* < 0.05, ** *p* < 0.01, *** *p* < 0.001.

The disruption of the intracellular calcium homeostasis triggers the ER stress response and vice versa [27]. Thus, we analyzed the relationship between EspC-induced ER stress and the release of intracellular Ca^2+^. EspC-induced intracellular Ca^2+^ release in the macrophages was significantly enhanced (Figure 3A, Fig S2C) but was inhibited by 4-PBA pretreatment (Figure 3B), implying that EspC-induced ER stress facilitates Ca^2+^ release from the ER. Moreover, the EspC-elicited cellular Ca^2+^ release was diminished following pretreatment with an intracellular Ca^2+^ chelator, BAPTA-AM (Figure 3C). Meanwhile, pre-incubation with BAPTA-AM reduced CHOP expression within EspC-induced RAW264.7 cells; similarly, caspase-3 activation and apoptosis decreased (Figure 3D, 3E). These results suggested that decreased cytosolic Ca^2+^ release suppressed EspC-triggered ER stress and apoptosis. Because BAPTA-AM can also block NF-κB activation [32], we further showed that the generation of pro-inflammatory cytokines elicited by EspC treatment was significantly repressed by BAPTA-AM in a dose-dependent manner (Figure 3F).

### EspC-mediated ER stress is associated with ROS production

**Figure 4.**
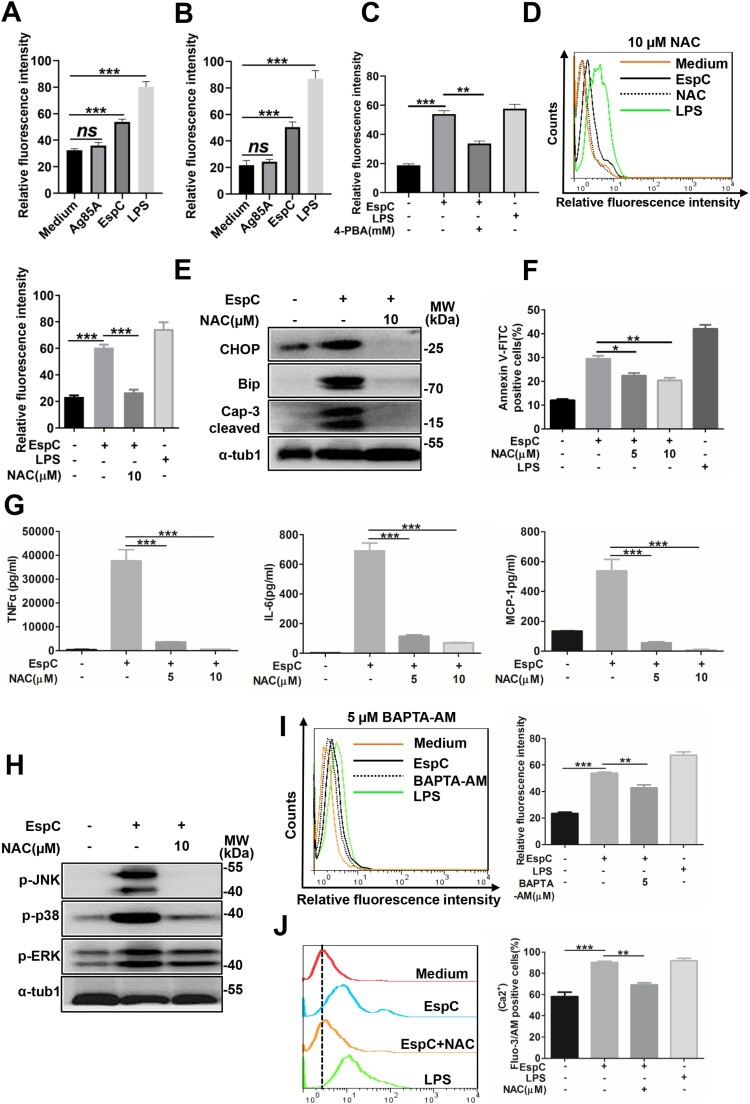
**EspC-mediated ER stress is associated with ROS production. (A, B)** Intracellular hydrogen peroxide **(A)** and superoxide **(B)** levels were evaluated using flow cytometry with DCFH-DA (10 µM) for hydrogen peroxide and dihydroethidium (DHE; 20 µM) for superoxide, after treatment with EspC or Ag85A for 24 h. LPS treatment was used as the positive control. **(C)** RAW264.7 cells were pretreated with 4-PBA (2 mM) for 1 h and then incubated with EspC for 30 h. Cells were collected and stained with DHE, following which superoxide production was detected using flow cytometry. **(D–G)** RAW264.7 cells were pretreated with NAC (5, 10 µM) for 1 h and then incubated with EspC for 30 h. **(D)** Cells were collected and stained with DHE, following which superoxide production was detected using flow cytometry. **(E)** Total cell lysates were subjected to western blot analysis with specific antibodies against each target protein. **(F)** Apoptotic cells were quantified using flow cytometry. **(G)** Pro-inflammatory cytokine (TNFα, IL-6, MCP-1) levels in the supernatant were measured using ELISA. **(H)** RAW264.7 cells were pretreated with NAC (10 µM) for 1 h and then incubated with EspC for 24 h. Total cell lysates were subjected to western blot analysis with specific antibodies against each target protein. **(I)** RAW264.7 cells were pretreated with BAPTA-AM (5 µM) for 1 h and then incubated with EspC (5 µg/mL) for 24 h. Cells were collected and stained with DHE, and superoxide production was detected using flow cytometry. **(J)** RAW264.7 cells were preincubated with or without NAC (10 µM) for 1 h, followed by EspC treatment for 30 h. The percentage of intracellular Ca^2+^ was measured with fluo-3/AM using flow cytometry. LPS treatment was used as the positive control to induce ER stress. Data are shown as the mean ± SEM (*n* = 3); * *p* < 0.05, ** *p* < 0.01, *** *p* < 0.001.

The enhanced generation of ROS and the rapid release of Ca^2+^ from the ER lumen are common features of the activation of cellular ER stress and unfolded protein response [33]. ROS can also elicit the production of pro-inflammatory cytokines via MAPK activation [34], and its accumulation has been reported to induce ER stress-related apoptosis during mycobacterial infection [30]. Here, ROS synthesis markedly increased after EspC treatment but was decreased following 4-PBA pretreatment (Figure 4A, B, S2D, S2E, C), suggesting that EspC-induced ER stress enhanced ROS generation. Moreover, pretreatment with N-acetyl-l-cysteine (NAC), a ROS scavenger, before EspC stimulation reduced ROS production and CHOP and Bip expression (Figure 4D, E). NAC treatment also decreased caspase-3 activation and alleviated apoptosis (Figure 4E, F). Furthermore, after NAC-preincubation, the production of TNFα, IL-6, and MCP-1 markedly reduced in a dose-dependent manner (Figure 4G), and the levels of phosphorylated p38, ERK, and JNK triggered by EspC were suppressed (Figure 4H). These results suggested that increased ROS can contribute to EspC-elicited ER stress and apoptosis and that sustained ROS generation further promotes inflammatory cytokine overload via MAPK activation [34].

Next, the link between intracellular Ca^2+^ dissipation and ROS generation was investigated. Following intracellular Ca^2+^ depletion using BAPTA-AM in RAW264.7 cells, ROS synthesis was significantly attenuated (Figure 4I). NAC treatment similarly decreased the release of intracellular Ca^2+^ (Figure 4J). These results suggested that EspC-mediated ER stress is associated with the cross-talk between ROS synthesis and intracellular calcium fluctuation.

### EspC-induced ER stress response is involved in mitochondrial apoptosis

**Figure 5.**
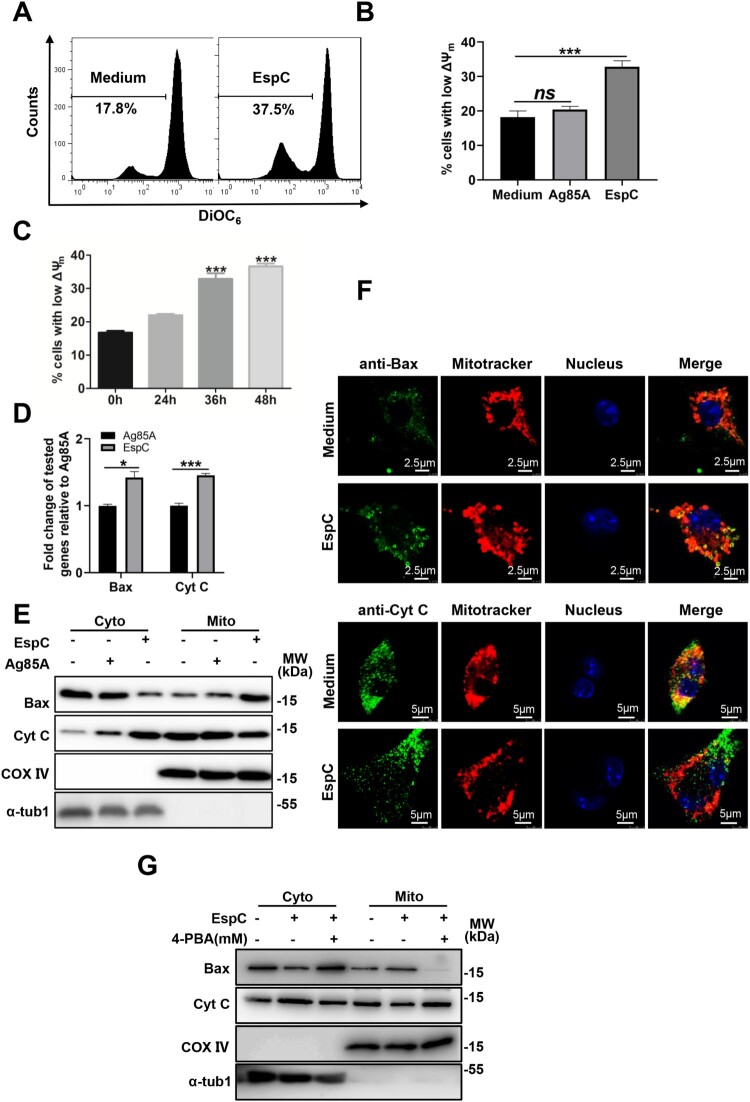
**EspC-induced ER stress response is involved in mitochondrial apoptosis. (A-C)** RAW264.7 cells were treated with PBS, EspC, or Ag85A for varying durations or 36 h. Cells were collected and stained with DiOC6 (10 nM), and ΔΨm was evaluated using flow cytometry **(D)** RAW264.7 cells were stimulated with EspC (5 µg/mL) or Ag85A for 24 h, and the expression levels of Bax and cytochrome c were analyzed using qPCR. **(E)** RAW264.7 cells were stimulated with EspC, Ag85A (5 µg/mL), or PBS for 36 h. Subcellular fractions of the mitochondria and cytoplasm were extracted and analyzed using western blot analysis with antibodies against Bax, cytochrome c (Cyt C), COX IV, and α-tub1. COX IV and α-tub1 were used as markers of mitochondrial and cytosolic fractions, respectively. **(F)** Representative confocal laser scanning microscopy images showing the Bax translocation from the cytosol into the mitochondria and the Cyt C release from the mitochondria to the cytosol in EspC-stimulated RAW264.7 cells. Scale bar = 5 µm or 2.5 µm. **(G)** RAW264.7 cells were pretreated with 4-PBA (2 mM) for 1 h and incubated with EspC for 36 h. Subcellular fractions of the mitochondria and cytoplasm were extracted and analyzed using western blot analysis with antibodies against Bax, cytochrome c (Cyt C), COX IV, and α-tub1. Data are shown as the mean ± SEM (*n* = 3); * *p* < 0.05, *** *p* < 0.001.

Numerous pro-death signals triggered by the ER stress response converge on the core mitochondrial apoptosis pathway [27]. We thus explored whether EspC affects the integrity and permeability of the mitochondria. Results of the flow cytometry analysis showed that the ΔΨm was significantly reduced in EspC-treated RAW264.7 cells (Figure 5A). Furthermore, the loss of ΔΨm was aggravated with time after EspC treatment (Figure 5B, C). The transcriptional levels of Bax and cytochrome c were enhanced in EspC-treated macrophages compared with those in Ag85A treatment (Figure 5D). Particularly, the level of cytosolic cytochrome c was enhanced, along with a reduced level within the mitochondrial fraction, and vice versa for Bax (Figure 5E). Meanwhile, the immunofluorescence assay yielded similar results (Figure 5F). Furthermore, the release of cytochrome c and Bax translocation was reduced when cells were pre-treated with 4-PBA (Figure 5G). Altogether, these results suggested that EspC-induced ER stress response is involved in the mitochondrial apoptosis pathway triggered by Bax translocation and cytochrome c release.

### Expression of Mtb EspC in Msm enhances ER stress-mediated apoptosis and promotes mycobacterial infection in macrophages and mice

**Figure 6.**
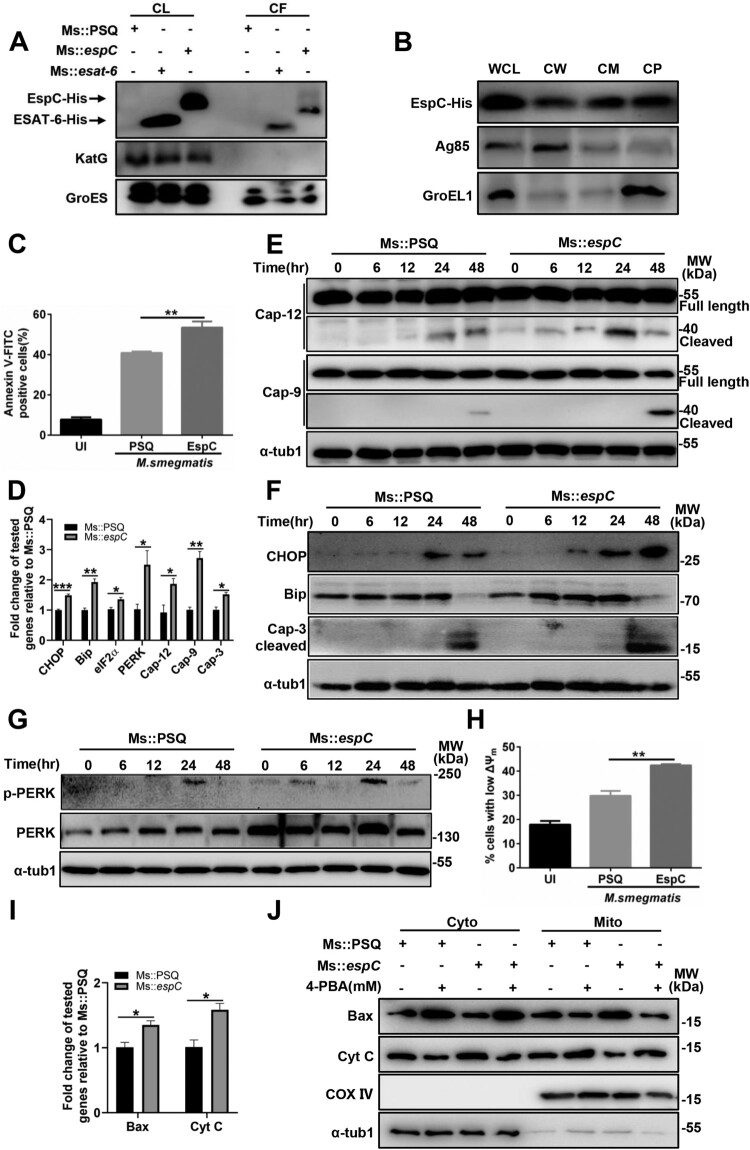
Expression of *Mtb* EspC in non-pathogenic *Msm* enhances ER stress-mediated apoptosis. **(A)** The indicated strains were cultured in Sauton’s medium containing 30 µg/mL kanamycin for 12 h, and the bacteria and cell-culture supernatant were harvested for western blot analysis using anti-His, anti-KatG, and anti-GroES antibodies. Ms::*esat-6* expressing and secreting ESAT-6 was used as the positive control. CL: supernatants of bacterial sonicated lysates; CF: cell culture filtrates. **(B)** Ms::*espC* were performed on fractionation experiments, and the isolated fractions were subjected to western blotting analysis using anti-His, anti-Ag85, and anti-GroEL1 antibodies. WCL: whole-cell lysates; CW: cell-wall fractions; CM: cell-membrane fractions; CP: cytoplasmic fraction. Cell wall Ag85 and cytoplasmic GroEL1 were used as the positive and negative controls, respectively. **(C–I)** RAW264.7 cells were infected with the indicated mycobacterial strains for the indicated durations at MOI = 10. **(C)** Apoptotic cells were quantified using flow cytometry. **(D)** The expression level of each target gene was analyzed using qPCR. **(E-G)** Total cell lysates were examined using western blot analysis with specific antibodies against each target protein. **(H)** Cells were collected and stained with DiOC6 (10 nM), and ΔΨm was evaluated using flow cytometry. **(I)** The expression levels of Bax and cytochrome c were determined using qPCR. **(J)** RAW264.7 cells were infected with the indicated mycobacterial strains for the indicated durations at MOI = 10 in the presence or absence 4-PBA. Subcellular fractions of the mitochondria and cytoplasm were extracted and analyzed using western blot analysis with antibodies against each target protein. Data are shown as the mean ± SEM (*n* = 3); * *p* < 0.05, ** *p* < 0.01, *** *p* < 0.001.

Given that the loss of EspC has a negative effect on the secretion of other virulence factors [15,16] and that EspC is not present in the *Msm*, we constructed recombinant *Msm* expressing the *Mtb* EspC (Ms::*espC*)[21] to investigate the function of EspC during host cell infection with mycobacteria. In general, the surface exposure or secretion into the extracellular environment allows Mtb proteins to interact directly with their host targets. We evaluated the secretion and localization of EspC in Ms::*espC*. The results showed that, similar to Mtb ESAT-6, EspC was present not only in whole-cell lysates but also in cell-culture fractions as secretions of Ms::*espC* (Figure 6A, S3A). The results of subcellular isolation assays showed that, aside from the cytoplasm, Mtb EspC was equally present in the cell membrane and cell wall of Ms::*espC* (Figure 6B). Moreover, after proteinase K treatment, the level of Mtb EspC in Ms::*espC* gradually decreased in a time-dependent manner (Fig S3B, S3C). These results suggested that Mtb EspC was secreted and surface exposed by Ms::*espC*.

The expression of *Mtb* EspC in *Msm* increased macrophage apoptosis (Figure 6C) and enhanced the transcriptional expression and activation of caspase-12, caspase-9, and caspase-3 (Figure 6D, E). In addition, Ms::*espC* improved the expression of ER-stress markers at both the mRNA and protein levels and the phosphorylation level of PERK (Figure 6D, F, 6G). Ms::*espC* also increased the amount of cells with low ΔΨm (Figure 6H). Consistently, the transcriptional levels of Bax and cytochrome c were increased (Figure 6I). Increased cytochrome c release and Bax translocation between the mitochondria and the cytosol were observed in macrophages infected with Ms::*espC* compared with the empty vector control (Ms::PSQ) (Figure 6J). Moreover, 4-PBA pretreatment reduced cytochrome c release and Bax translocation in Ms::*espC*-infected macrophages (Figure 6J). Overall, these results indicate that the *Mtb* ESX-1 protein EspC is sufficient to promote mycobacteria to induce ER stress-mediated apoptosis.

**Figure 7.**
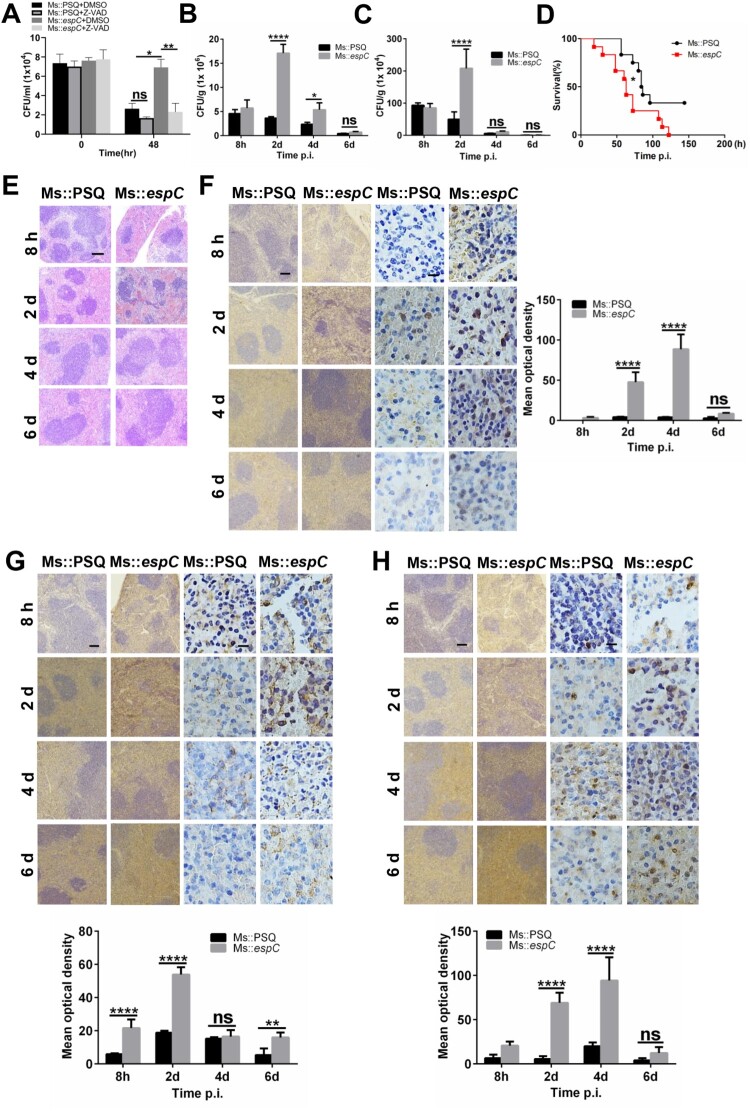
**EspC induces ER stress and apoptosis and promotes mycobacterial infection in mice. (A)** Intracellular survival of *Msm* in RAW264.7 cells infected with Ms::PSQ and Ms::*espC* at MOI = 10 for the indicated time in the absence or presence of Z-VAD (50 µM). **(B, C)** C57BL/6 mice were challenged with Ms::PSQ or Ms::*espC* (1 × 10^6^ CFU/mouse), and CFUs were counted in the spleens **(B)** and lungs **(C)** of the infected mice at 8 h, and days 2, 4, and 6 post-infection. **(D)** Survival of WT C57BL/6 mice after infection with Ms::PSQ or Ms::*espC* (1 × 10^7^ CFU/mouse) administered via i.v. injection (* *p* < 0.05, Gehan–Breslow–Wilcoxon Test). **(E)** C57BL/6 mice were challenged with Ms::PSQ or Ms::*espC* (1 × 10^6^ CFU/mouse), and hematoxylin and eosin (HE) staining was performed in the spleen tissues of infected mice at indicated times at 8 h, and days 2, 4, and 6 post-infection. Scale bar = 100 µm. **(F–H)** Immunostaining of CHOP **(F)**, Bip **(G),** and cleaved caspase-3 **(H)** was performed on the spleen tissues of infected mice. Scale bar = 100 µm or 10 µm. The bar plot shows the CHOP, Bip, or cleaved caspase-3 intensity (mean optical density) in the spleen. Data are shown as the mean ± SEM (*n* = 3); * *p* < 0.05, ** *p* < 0.01, *** *p* < 0.001.

We have previously found that EspC-overexpression increased the survival of *Msm* within macrophages [21]. To determine whether the enhanced intracellular survival of mycobacteria is due to EspC-mediated apoptosis, we evaluated the survival of Ms::*espC* in macrophages upon apoptosis inhibition by Z-VAD*.* We found that Z-VAD pretreatment significantly decreased the intracellular survival of Ms::*espC* in macrophages in comparison with those of Ms::PSQ 48 h post-infection (Figure 7A), suggesting that EspC-mediated apoptosis is beneficial to the intracellular survival of mycobacteria.

Lastly, to investigate whether EspC is involved in the survival of mycobacteria *in vivo*, WT C57BL/6 mice were intravenously infected with Ms::PSQ or Ms::*espC* using an acute mycobacterial infection model. Compared with Ms::PSQ, the EspC-overexpressed *Msm* exhibited markedly increased bacterial load, with 5- and 2.5-fold higher CFUs in mouse spleens 2 and 4 days post-infection, respectively (Figure 7B). Compared with Ms::PSQ, the EspC-overexpressing *Msm* showed significantly increased bacterial load, with 4-fold higher CFU in mouse lungs 2 days post-infection (Figure 7C). Consistently, Ms::*espC* infection significantly reduced mouse survival in comparison with Ms::PSQ (Figure 7D). Ms::*espC* caused increased infiltration of myeloid cells with the expansion of white pulp and involution of red pulp in comparison with Ms::PSQ-infected mice (Figure 7E). Meanwhile, immunohistochemistry staining results showed that infection with Ms::*espC* significantly increased the expression of the ER-stress markers CHOP (Figure 7F) and Bip (Figure 7G) and the levels of cleaved caspase-3 (Figure 7H) in mouse spleen compared with infection with Ms::PSQ. Taken together, our results suggested that the apoptosis mediated by EspC-induced ER stress may contribute to mycobacterial infection in mice.

## Discussion

Increasing evidence has shown that ESX-1 components exhibit distinctive effects on the secretion of ESX-1-dependent substrates [35–37]. Thus, this raises the question of whether only EsxA in the ESX-1 system plays a role in mycobacterial virulence and interaction with host cells. EspC is highly conserved in pathogenic mycobacteria [15,16] and actively expressed during *Mtb* infection *in vivo* [38], indicating the possibility of an interaction between EspC and the macrophages and pathogenecity. We have previously confirmed that *Mtb* EspC activates macrophages through the TLR4-dependent MAPK signaling pathway and enhances the survival of mycobacteria in host cells [21]. Here, we demonstrate how EspC triggers the ER stress-mediated apoptosis of macrophages and enhances mycobacterial infection in mice.

**Figure 8.**
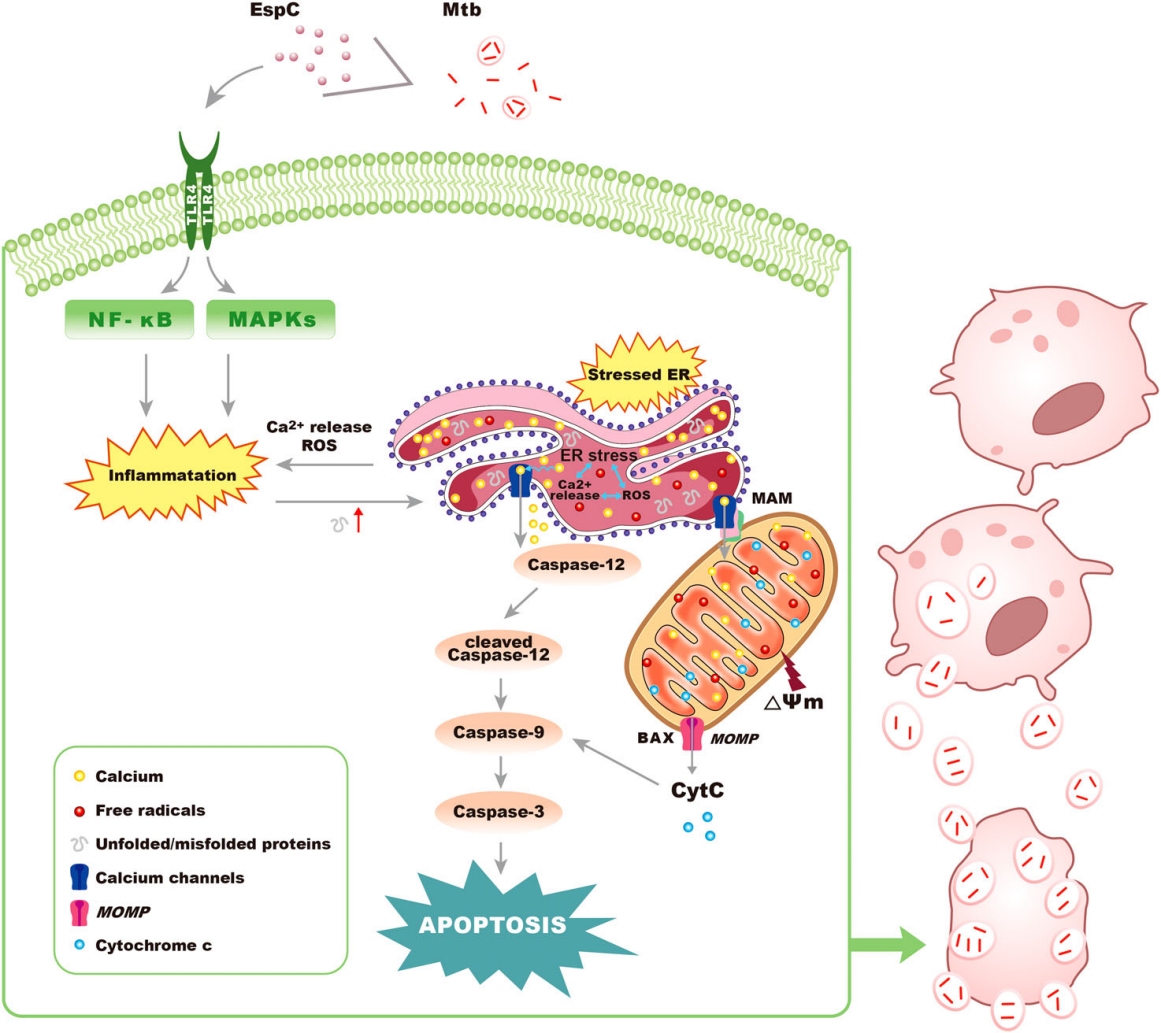
Proposed mode of action of *Mtb* EspC. *Mtb* EspC-induced pro-inflammatory cytokine overload through MAPK/ NF-κB cascades initiates the ER stress response, leading to intracellular calcium disturbance and ROS generation. This subsequently results in the generation of more pro-inflammatory cytokines, thus aggravating the ER stress. The cross-talk and interactions among ER stress, intracellular calcium fluctuations, ROS production, and pro-inflammatory cytokine overload trigger a sustained ER stress response and eventually cause apoptosis, which might promote mycobacterial infection. MOMP, mitochondrial outer membrane permeabilization; MAM, mitochondrial associated membrane.

ER stress is caused by the accumulation of unfolded or misfolded proteins in the ER, and if not alleviated, it can activate downstream signaling pathways that induce cell apoptosis [27]. ER stress is induced in the macrophages of granulomas as a result of *Mtb* infection [28]. Here, we found that the *Mtb* EspC induced the ER stress-mediated apoptosis via unfolded-protein response. Chronic ER stress and the unfolded-protein response activation resulted in impaired calcium and redox homeostasis [33]. Consistently, EspC stimulation increased intracellular Ca^2+^ release and ROS accumulation and improved ER-folding capacity by 4-PBA attenuated EspC-mediated Ca^2+^ release and ROS generation. Moreover, the ER stress and apoptosis caused by EspC were attenuated when intracellular Ca^2+^ release and ROS generation were decreased. In addition, we found that treatment with ROS scavengers mitigated EspC-induced Ca^2+^ release and in turn Ca^2+^ chelators attenuated ROS accumulation. The release of intracellular Ca^2+^ affects the activities of calcium-dependent enzymes and chaperones in the ER, causing the accumulation of unfolded proteins and resulting in ER stress [27,33]. In addition, Ca^2+^ chelators inhibited ROS formation depending on Ca^2+^ efflux [33], and ROS can target ER resident proteins and Ca^2+^ channels, thus leading to the release of Ca^2+^ and ER-stress signaling molecules [33]. The inactivation of NF-κB and MAPK pathways relieved the EspC-mediated ER stress, thereby supporting the accelerated generation of pro-inflammatory cytokines and leading to the accumulation of unfolded or misfolded proteins [39]. Moreover, we found that either ER-folding capacity improved by 4-PBA treatment or preincubation with Ca^2+^ chelator, such as BAPTA-AM, or antioxidant-like NAC suppressed the EspC-induced production of pro-inflammatory cytokines and alleviated ER stress. Consistently, sustained ER stress caused by the accumulation of unfolded proteins is known to elicit inflammatory responses [40], and ER stress-mediated activation of NF-κB and MAPK depends on the ER Ca^2+^ efflux and ROS formation [40,41]. The close link between ER stress and inflammation is likely to be a factor in the integration between ER function and cell fate, and they are destructive and go beyond physiological control when chronically induced [40]. Thus, we propose that EspC, a novel pro-apoptotic factor, can induce pro-inflammatory cytokine overload through MAPK/NF-κB cascades and initiate the ER stress response, leading to intracellular calcium disturbances and ROS generation, which in turn results in the synthesis of more pro-inflammatory cytokines, thereby aggravating the ER stress. Therefore, the cross-talk and interactions involved in ER stress, intracellular calcium fluctuations, ROS production, and pro-inflammatory cytokine overload trigger a sustained ER stress response and the eventual apoptosis (Figure 8).

The subject of apoptotic roles during infection is widely discussed. However, the observations and interpretation of data in different experimental settings seem to be rather heterogenic. Previous reports have suggested that apoptosis is a macrophage defense process for the suppression of *Mtb* infection [42]. The virulent *Mtb* strains have can induce the apoptosis of host cells [11,30,43], and alveolar macrophages and caseating granulomas from patients with pulmonary TB also exhibit apoptosis-characterized cell death [44,45]. In addition, ER stress and apoptotic markers have been found in both infected lungs and macrophages, indicating that these signaling routes are activated by *Mtb* and affect TB pathogenesis [28,30]. Apoptosis could have dual and opposing roles during infection, in which both the host and pathogen aim to use this process for their own benefit. Increasing evidence supports that apoptosis mediated by *Mtb*-secreted proteins facilitates bacterial pathogenesis. Several studies have shown that ESAT-6-secreting strains, such as H37Rv (but not H37Ra), RD1-defective mutant H37Rv, and BCG, induced apoptosis in macrophages and mice [11,43]. Additionally, ESAT-6-mediated, ER stress-induced apoptosis enhanced the intracellular survival of ESAT-6-secreting strains both *in vitro* and *in vivo* [43,46], suggesting that *Mtb* ESX-1 might induce apoptosis in host phagocytes as a bacterial spread strategy [47]. Moreover, *Mtb* H37Rv induced an ER stress response through the eIF2α/CHOP pathway to enhance the intracellular survival of H37Rv [30]. In addition, the Rv0297-encoded PE_PGRS5 protein present in the later stages of human granulomas could localize to the ER and induce the unfolded protein response and the subsequent apoptosis of host cells, thus supporting the ER stress-mediated apoptosis, which might also benefit the dissemination of bacteria in advanced granulomas.

Here, we showed that EspC-treated macrophages triggered ER stress-induced, mitochondria-mediated apoptosis rather than necrosis. Mitochondrial damage plays a crucial role in the outcome of macrophage infection with *Mtb* [42,48]. EspC-mediated apoptosis enhanced the intracellular survival of EspC-expressing strains in both *in vitro* and *in vivo* conditions. Moreover, decreased apoptosis following treatment with the inhibitor Z-VAD significantly reduced the intracellular survival of Ms::*espC* in macrophages*.* These findings support the idea that apoptosis is a bacterial infection mechanism. This has been previously described in zebrafish infected with *M. marinum*, which caused apoptosis in an ESX-1-dependent manner. In this previous study, the apoptotic infected macrophages were engulfed by motile macrophages, leading to the spread of disease beyond the primary granuloma [49]. A similar infection mechanism has been described for *Yersinia pestis*, the etiological agent of the plague, which survives in human neutrophils. When they undergo apoptosis, they are engulfed by macrophages [50], suggesting that some pathogens have successfully adapted to and circumvented host defense mechanisms. These pathogens have taken advantage of the engulfment of apoptotic infected cells in a process called efferocytosis to survive and disperse or disseminate.

However, the exact role of the ESX-1 individual substrates is difficult to elucidate via original knockout strains of an indicated gene owing to their complicated interdependency during secretion, such as with EspC and EsxA. In other words, EspC-deletion mutant abolishes the secretion of EsxA; conversely, EsxA secretion-defective mutants are defective for secretion of EspC and several other ESX-1 substrates. Although there is no homologous *espC* in the *Msm* genome, homologous MsESAT-6 or ESAT-6/CFP-10 proteins from Mtb were still secreted via the endogenous ESX-1 system in *Msm* (Figure 6A)[24]. *Converse et. al* reported that the secretion of MsESAT-6, MsCFP-10, Mtb ESAT-6, and CFP-10 homologues is strictly dependent on the *Msm* Snm machinery [24]. Whether Mtb EspC is also secreted by the endogenous Snm system in Ms::*espC* warrants further studies.

Considering the prominent roles of bacterial secretion systems in immunity and pathogenesis, we believe our findings provide a new perspective to understand the complex roles of virulent factors in host–pathogen interactions and the mechanism underlying ESX-1-mediated pathogenesis. This could help predict candidates for future TB vaccines and therapeutic targets for *Mtb* infection.

## Supplementary Material

Table_S2_final.xlsx

Table_S1_final.xlsx

Supplementary_Materials__For_Revision_final.docx

## Data Availability

Data are available via ProteomeXchange with identifier PXD013666.
