# Supplementary material for: Mycobacterium tuberculosis ESX-1-secreted substrate protein EspC promotes mycobacterial survival through endoplasmic reticulum stress-mediated apoptosis
Source: Emerg Microbes Infect. 2021 Jan 17;10(1):19–36. doi: 10.1080/22221751.2020.1861913 (PMC7832037; doi:10.1080/22221751.2020.1861913)
Supplement: Supplementary_Materials__For_Revision_final.docx [file TEMI_A_1861913_SM5553.docx]

**Supplementary Materials**

***Mycobacterium tuberculosis* ESX-1-secreted substrate protein EspC promotes mycobacterial survival through endoplasmic reticulum stress-mediated apoptosis**

Qinglong Guo, Jing Bi, Honghai Wang, Xuelian Zhang

**Correspondence:** Dr. Xuelian Zhang: xuelianzhang@fudan.edu.cn

**Supplementary Materials consists of 4 tables (Table S1-S4), 3 figures (Figure S1-S3), and Supplementary Figure Legends.**

| **Table S3. Quantified proteins involved in different signaling pathways.** | | | | | | |
| --- | --- | --- | --- | --- | --- | --- |
| **Uniprot ID** | **Gene symbol** | **Protein description** | **Coverage** | **Unique peptide** | **Abundance ratio: (F1, sample)/(F1, control)** | **p-value** |
| **Protein processing in the endoplasmic reticulum and endoplasmic reticulum stress response** | | | | | | |
| Q03963 | Eif2ak2 | Interferon-induced, double-stranded RNA-activated protein kinase | 22.718 | 10 | 1.448 | 2.89E-06 |
| P48722 | Hspa4l | Heat shock 70 kDa protein 4L | 24.821 | 17 | 1.392 | 0.019756 |
| Q4FJZ3 | Ero1l | Ero1l protein | 26.939 | 9 | 1.312 | 0.000284 |
| P20029 | Hspa5 (Bip) | 78 kDa glucose-regulated protein | 56.335 | 38 | 1.309 | 5.72E-06 |
| **Response to oxidative stress** | | | | | | |
| Q3U125 | Fam213a | Putative uncharacterized protein | 19.651 | 5 | 1.728 | 8.74E-06 |
| Q4FJZ3 | Ero1l | Ero1l protein | 26.940 | 9 | 1.312 | 0.000284 |
| **Peroxisome** | | | | | | |
| P99029 | Prdx5 | Peroxiredoxin-5, mitochondrial | 43.810 | 9 | 1.737 | 0.000332 |
| Q4FJX9 | Sod2 | Superoxide dismutase | 22.973 | 5 | 1.389 | 0.001325 |
| **MAPK and NF-**κ**B signaling pathway** | | | | | | |
| Q05769 | Ptgs2 | Prostaglandin G/H synthase 2 | 18.709 | 8 | 1.742 | 1.91E-05 |
| Q6Q899 | Ddx58 | Probable ATP-dependent RNA helicase DDX58 | 12.311 | 13 | 1.598 | 7.91E-06 |
| A0A087WSP5 | Stat1 | Signal transducer and activator of transcription | 19.868 | 15 | 1.572 | 1.71E-05 |
| Q52L50 | Rap1b | Putative uncharacterized protein | 37.5 | 2 | 1.333 | 0.001584 |
| **Apoptosis** | | | | | | |
| P06797 | Ctsl | Cathepsin L1 | 19.162 | 6 | 1.486 | 0.000249 |
| Q8BVK9 | Sp110 | Sp110 nuclear body protein | 25.618 | 5 | 1.381 | 0.00178 |
| Q5NBU8 | Xaf1 | XIAP-associated factor 1 | 9.524 | 2 | 1.312 | 0.003212 |

**Table S4. Primers of each target gene for qPCR.**

| **Oligonucleotides (5'-3')** | | |
| --- | --- | --- |
| CHOP | sense | CATGAACAGTGGGCATCACC |
|  | anti-sense | GCTGGGTACACTTCCGGAGAG |
| Bip | sense | CACGTCCAACCCCGAGAA |
|  | anti-sense | ATTCCAAGTGCGTCCGATG |
| eIF2α | sense | ATGGAAGCCAAAGCTGAAG |
|  | anti-sense | CTGACATGAAGGAGGGCA |
| PERK | sense | CCGCAAGAAGGACCCTATCC |
|  | anti-sense | GAGTTTCAGACTCCTTCCGCT |
| Bax | sense | AGGATGATTGCTGACGTGGA |
|  | anti-sense | AGCCACCCTGGTCTTGGA |
| cytochrome c | sense | GAGGCAAGCATAAGACTGGA |
|  | anti-sense | TACTCCATCAGGGTATCCTC |
| caspase-12 | sense | CTCTAACTGTCGGAGTCTGAGAAA |
|  | anti-sense | TCAGCAGTGGATATCCCTTTG |
| caspase-9 | sense | CAAGTTTGCCTACCCCCAGT |
|  | anti-sense | AACCCTGAGAAGGAGGGACT |
| caspase-3 | sense | TCATCTCGCTCTGGTACGGA |
|  | anti-sense | ACACACACAAAGCTGCTCCT |
| GAPDH | sense | ATGTTCCAGTATGACTCCACTCACG |
|  | anti-sense | GAAGACACCAGTAGACTCCACGACA |

**A**

**B**

**Figure S1**


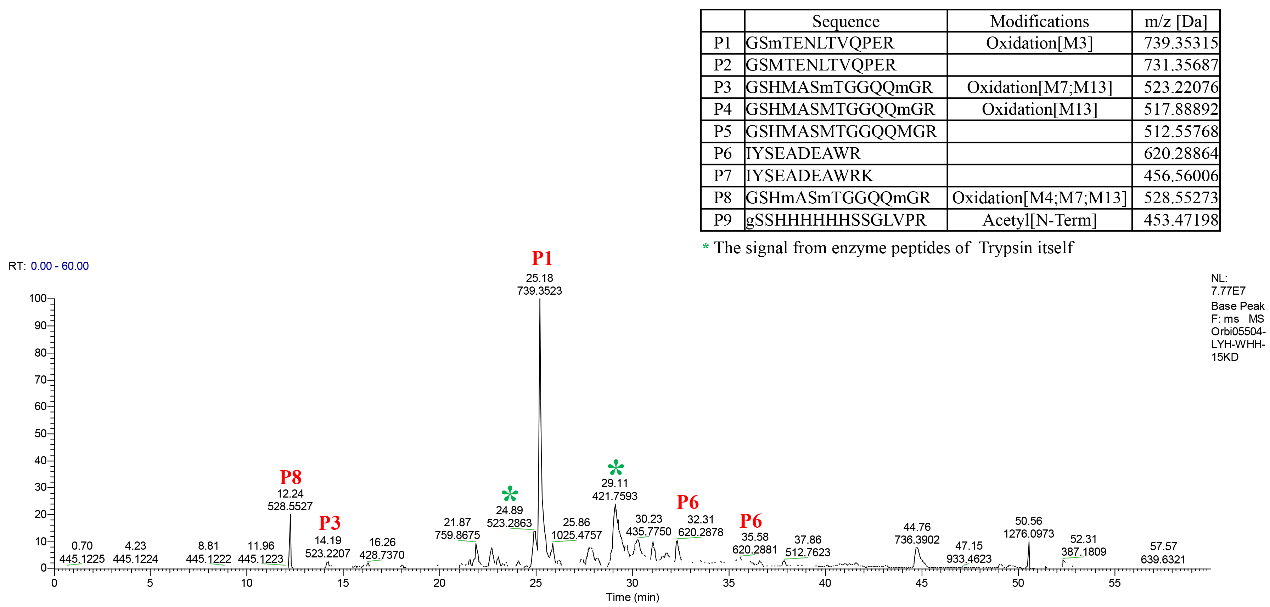


**C**


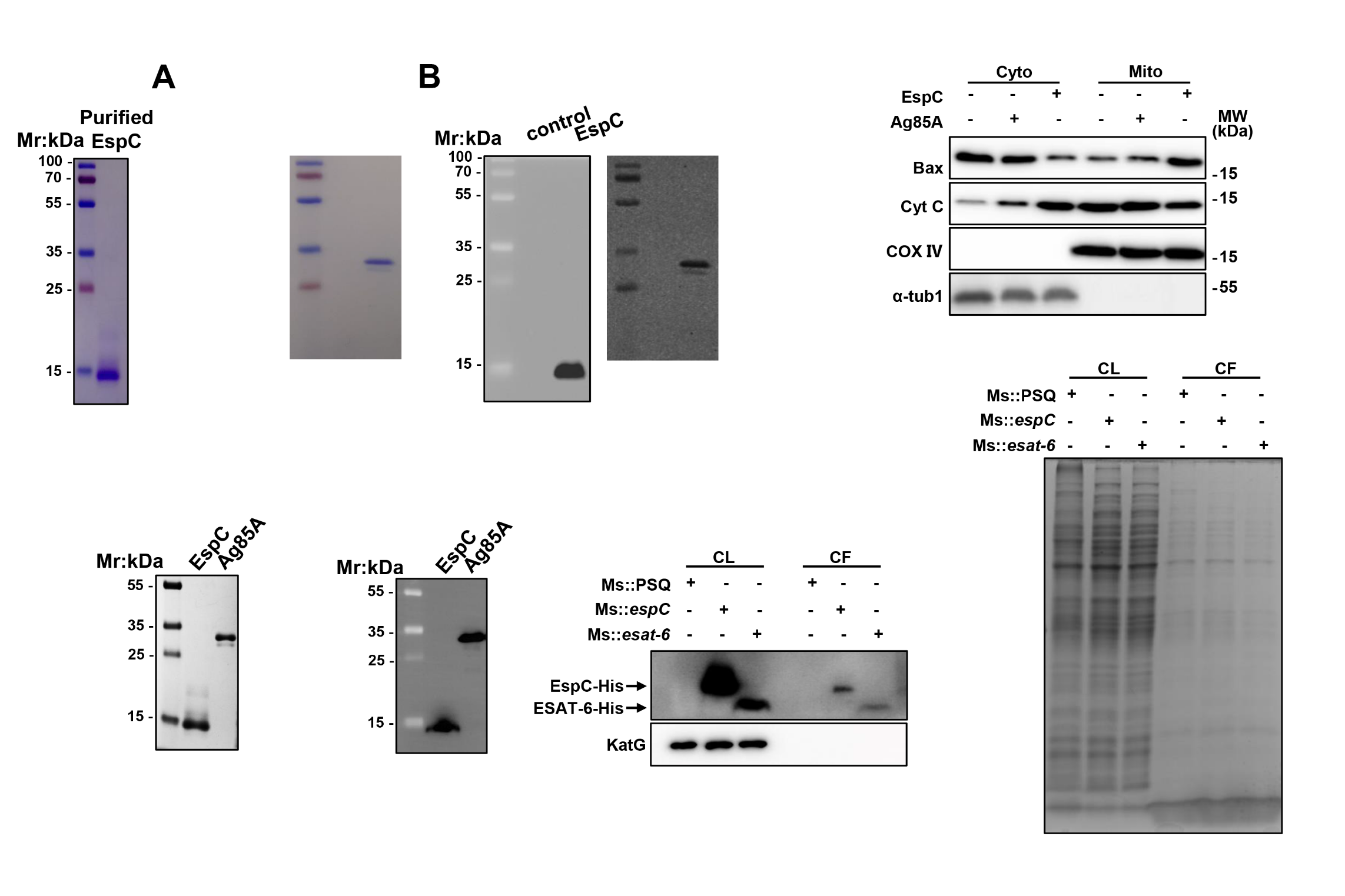

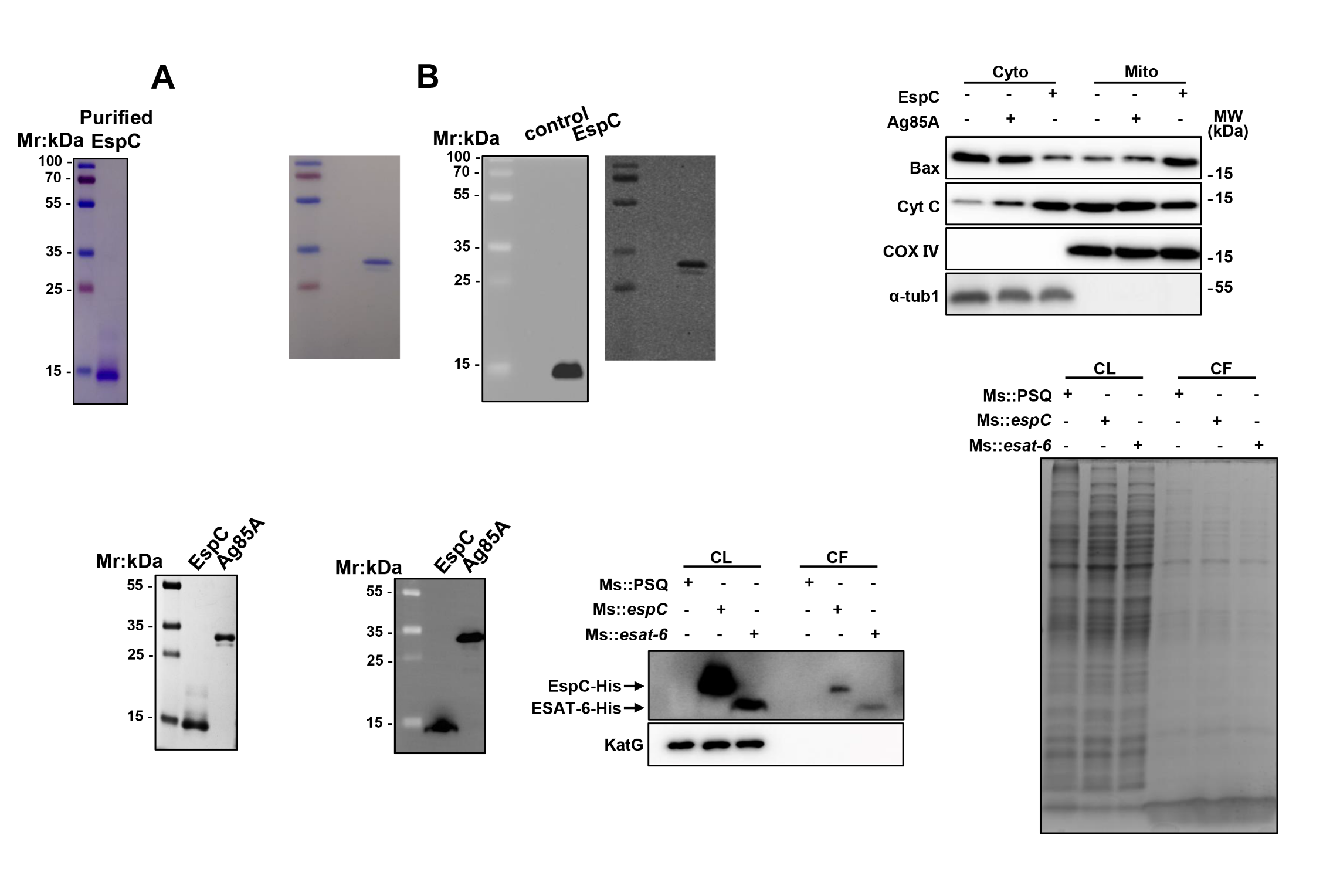


**Figure S2**

**A**

**B**


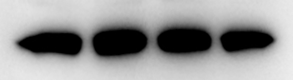

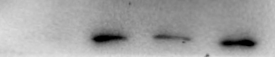


**Medium**

**EspC**

**Ag85A**

**LPS**

**Cap-9**

**Cap-3**

**Cap-12**


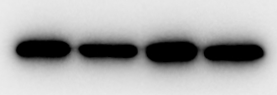

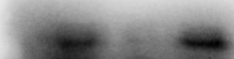

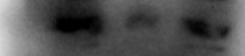


**MW**

**(kDa)**

**Full length**

**Full length**

**Cleaved**

**Cleaved**

**Full length**

**Cleaved**

**­-55**

**­-40**

**­-55**

**­-40**

**­-35**

**­-15**

**­-55**

**α-tub**


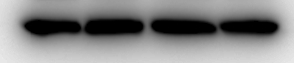

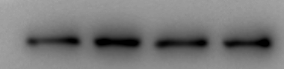

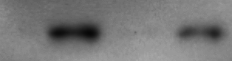


**Medium**

**EspC**

**Ag85A**

**LPS**

**MW**

**(kDa)**

**p-eIF2α**

**CHOP**

**Bip**

**α-tub1**

**­-25**

**­-35**

**­-70**

**­-55**

**­-40**


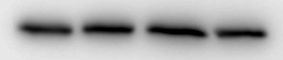

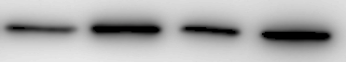

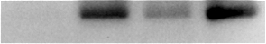


**C**

**D**

**E**


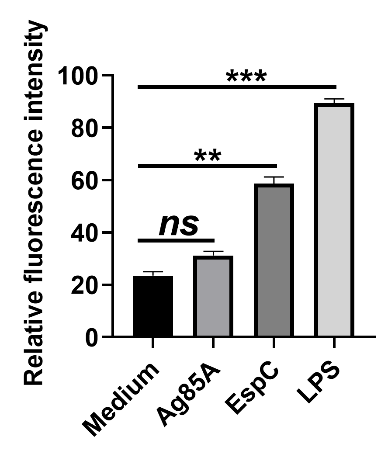

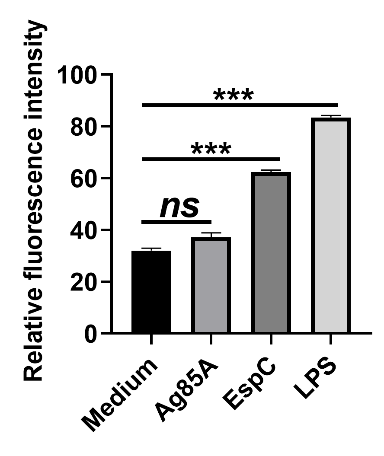

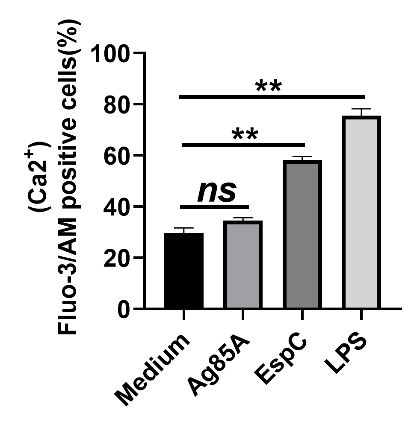


**Figure S3**

**C**

**B**

**A**


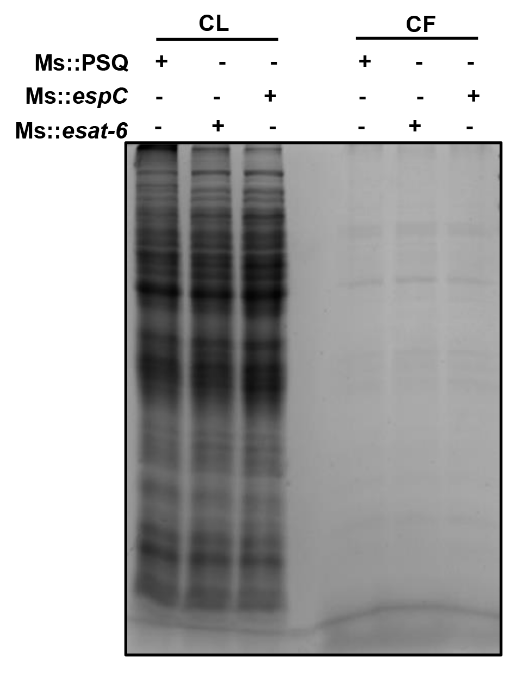


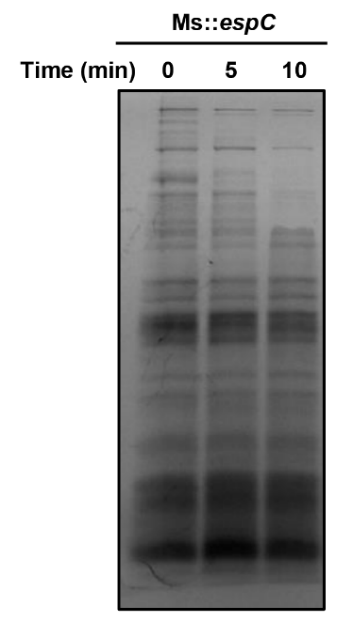


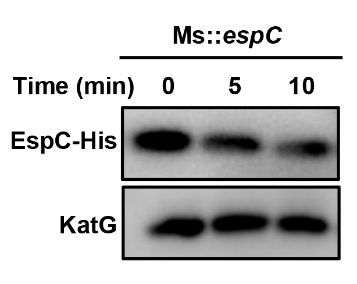


**Supplementary Figure Legends**

**Table S1.** Proteins identified using iTRAQ-based quantitative proteomics.

**Table S2.** Differentially expressed proteins (Abundance Ratio: (F1, sample)/(F1, control) >1.3 or <0.7; *p* < 0.05) identified using iTRAQ-based quantitative proteomics.

**Table S3.** Quantified proteins involved in different signaling pathways.

**Table S4.** Primers of each target gene for qPCR.

**Figure S1**. **Expression and purification of recombinant EspC and Ag85A proteins.** (A-C) Protein was expressed in *Escherichia coli* and purified using Ni-NTA affinity chromatography. The purified protein was subjected to SDS-PAGE analysis by staining with Coomassie blue (A) or western blot analysis using anti-His mouse antibodies (B) or LC–MS analysis (C). Ag85A was used as the negative control expressed and purified similarly.

**Figure S2. Effects of EspC on macrophages.** (A) Immunoblot analysis of CHOP, Bip, p-eIF2α, and α-tubulin (α-tub1) in the cell lysates of RAW264.7 cells stimulated with EspC or Ag85A (equal molar) for 24 h. (B) RAW264.7 cells were treated with EspC or Ag85A (equal molar) for 24 h, and the cell lysates were examined using western blot analysis with antibodies against caspase-12 (Cap-12), caspase-9 (Cap-9), caspase-3 (Cap-3), and α-tub1. (C) RAW264.7 cells were stimulated with EspC or Ag85A (equal molar) for 24 h. The percentage of increased intracellular Ca^2+^ levels was measured with fluo-3/AM using flow cytometry. (D, E) Intracellular hydrogen peroxide **(A)** and superoxide **(B)** levels were evaluated using flow cytometry with DCFH-DA (10 µM) for hydrogen peroxide and dihydroethidium (DHE; 20 µM) for superoxide, after treatment with EspC or Ag85A (equal molar) for 24 h.

**Figure S3. expression and subcellular localization of EspC in *M. smegmatis*.** (A) The indicated strains were cultured in Sauton’s medium containing 30 µg/mL kanamycin for 12 h, and the bacteria and cell-culture supernatant were harvested for SDS-PAGE analysis. Ms::*esat-6* expressing and secreting ESAT-6 was used as the positive control. CL: supernatants of bacterial sonicated lysates; CF: cell culture filtrates. (B, C) Ms::*espC* was digested by proteinase K for 0, 5, and 10 min. The samples were subjected to western blotting analysis using anti-His and anti-KatG antibodies (B) and SDS-PAGE analysis (C). Cytosolic KatG was used as the negative control.
